# IbOr Regulates Photosynthesis under Heat Stress by Stabilizing IbPsbP in Sweetpotato

**DOI:** 10.3389/fpls.2017.00989

**Published:** 2017-06-08

**Authors:** Le Kang, Ho S. Kim, Young S. Kwon, Qingbo Ke, Chang Y. Ji, Sung-Chul Park, Haeng-Soon Lee, Xiping Deng, Sang-Soo Kwak

**Affiliations:** ^1^Plant Systems Engineering Research Center, Korea Research Institute of Bioscience and BiotechnologyDaejeon, South Korea; ^2^Department of Green Chemistry and Environmental Biotechnology, Korea University of Science and TechnologyDaejeon, South Korea; ^3^Environmental Biology and Chemistry Center, Korea Institute of ToxicologyJinju, South Korea; ^4^State Key Laboratory of Soil Erosion and Dryland Farming on the Loess Plateau, Institute of Soil and Water Conservation, Northwest A&F UniversityShaanxi, China

**Keywords:** chaperone activity, heat stress, *IbOr*, *IbPsbP*, sweetpotato

## Abstract

The Orange (Or) protein regulates carotenoid biosynthesis and environmental stress in plants. Previously, we reported that overexpression of the sweetpotato [*Ipomoea batatas* (L.) Lam] *Or* gene (*IbOr*) in transgenic *Arabidopsis* (referred to as *IbOr*-OX/At) increased the efficiency of photosystem II (PSII) and chlorophyll content after heat shock. However, little is known about the role of IbOr in PSII-mediated protection against abiotic stress. In this study, comparative proteomics revealed that expression of PsbP (an extrinsic subunit of PSII) is up-regulated in heat-treated *IbOr*-OX/At plants. We then identified and functionally characterized the *PsbP*-*like* gene (*IbPsbP*) from sweetpotato. IbPsbP is predominantly localized in chloroplast, and its transcripts are tissue-specifically expressed and up-regulated in response to abiotic stress. In addition, IbOr interacts with IbPsbP and protects it from heat-induced denaturation, consistent with the observation that transgenic sweetpotato overexpressing *IbOr* maintained higher PSII efficiency and chlorophyll content upon exposure to heat stress. These results indicate that IbOr can protect plants from environmental stress not only by controlling carotenoid biosynthesis but also by directly stabilizing PSII.

## Introduction

Abiotic stresses, including extreme temperatures, drought, salinity, and oxidative stress, are serious threats to agriculture and cause deterioration of the environment. Given the dramatic increase in global population and serious global environmental problems (Tilman et al., 2001), the development of high-nutrient, abiotic stress tolerant crops is an urgent requirement for a sustainable society. However, current breeding techniques (i.e., traditional crossbreeding or ectopic expression of transcription factors) have become bottlenecks, creating severe impediments to crop improvement (Akhond and Machray, 2009). Thus, genetic modification technologies are needed to improve crop sustainability and productivity (Qaim and Zilberman, 2003).

In plants, carotenoids known as antioxidant play essential roles in light-harvesting processes and protect the photosynthetic machinery from photo-oxidative damage. The Orange (Or) protein, which functions as a holdase chaperone, post-transcriptionally regulates phytoene synthase (PSY), the rate-limiting enzyme in the carotenoid biosynthetic pathway (Zhou et al., 2015; Park et al., 2016). IbOr is mainly localized in the nucleus, and IbOr localization prominently changes to the chloroplast in response to heat stress (Park et al., 2016). IbOr holdase chaperone activity protects IbPSY stability, which leads to carotenoid accumulation, and confers enhanced heat and oxidative stress tolerance in plants (Park et al., 2016). The *Or* gene was first identified as a gain-of-function mutant allele that accelerates the formation of chromoplasts, creating a metabolic sink for carotenoid accumulation in cauliflower (Li et al., 2006; Lu et al., 2006). *Or* genes are highly conserved in other species, including *Arabidopsis* (*AtOr*), *Brassica oleracea* (*BoOr*), and sweetpotato (*IbOr*). The Or protein contains an N-terminal region required for interaction with PSY and a C-terminal cysteine-rich zinc finger domain found in DnaJ-like molecular chaperones (Lu et al., 2006; Zhou et al., 2015; Park et al., 2016). Furthermore, our previous results suggested that IbOr translocates from the nucleus to the chloroplast in response to heat stress (Park et al., 2016). These findings suggest that Or is a multi-functional protein. Interestingly, recent work showed that transgenic sweetpotato calli, *Arabidopsis*, alfalfa, and potato overexpressing *IbOr* accumulate carotenoids and maintain higher photosystem II (PSII) efficiency and chlorophyll content under abiotic stress (Kim et al., 2013; Park et al., 2015, 2016; Wang et al., 2015; Cho et al., 2016). However, the mechanisms underlying the effects of Or on carotenoid biosynthesis and the abiotic stress response are currently unknown.

Photosystem II is a multisubunit membrane protein complex that performs light-induced electron transfer and water-splitting reactions, leading to the formation of molecular oxygen. In higher plants, PSII plays an especially important role in the response of photosynthesis to environmental perturbations and stresses (Havaux, 1992). PsbP is an extrinsic 23 kDa subunit of the oxygen-evolving complex (OEC) of PSII (Kochhar et al., 1996). On the luminal side of PSII, a cluster of three inorganic ions, manganese (Mn), calcium (Ca), and chloride (Cl), catalyze water oxidation. *In vitro* and *in vivo* studies showed that PsbP is required to maintain the active Mn-Ca-Cl cluster, and is essential for the water-splitting reaction (Ifuku et al., 2008). PsbP, a nuclear-encoded protein, is highly conserved in higher plants. Numerous studies showed that there are several homologs of the PsbP protein family in model plants such as *Arabidopsis* and tobacco (Ishihara et al., 2007; Yi et al., 2009). *PsbP* knockdown by RNA interference in *Arabidopsis* and tobacco revealed that PsbP proteins are essential for regulation and stabilization of PSII. In addition, the active form of the PSII light-harvesting complex II supercomplex plays distinct roles in photosynthetic electron transfer, and is required for normal thylakoid architecture in higher plants (Ishihara et al., 2007; Yi et al., 2007, 2008; Ido et al., 2009; Ifuku et al., 2011). Beyond those normal functions, PsbP may also influence the carotenoid or strigolactone biosynthetic pathways, as well as other physiological processes (Meier et al., 2011; Bricker et al., 2013). However, the molecular mechanisms underlying PsbP function (e.g., the mode of binding to PSII or other regulators) have not been elucidated.

Heat stress is one of the main abiotic stresses that limit the growth and productivity of plants (Boyer, 1982). Photosynthesis is most sensitive to such heat stress among various physiological processes (Berry and Björkman, 1980). Among various machineries of photosynthesis, PSII is particularly sensitive to heat, and even a short period of exposure to high temperatures irreversibly inactivates the OEC of PSII (Enami et al., 1994). Heat stress to chloroplasts caused the release of PsbO, PsbP, and PsbQ proteins and loss of cofactors. Loss of cofactors, especially PsbU, induced the inactivation of PSII, thermal damage of D1 protein, and production of ROS both in light and in dark when thermal stress persists (Balint et al., 2006). It was suggested that protection of the oxygen-evolving machinery by the extrinsic proteins of PSII was shown essential for thermotolerance (Allakhverdiev et al., 2008). On the other hand, PsbO and PsbP proteins are up-regulated by heat stress in barley leaf (Rollins et al., 2013). Until now, the precise mechanism of heat inactivation of PSII is not completely explained.

In this study, to investigate the overall roles of Or protein in photosynthesis and the abiotic stress response, we identified IbPsbP as an IbOr-interacting protein and confirmed its role in transgenic sweetpotato in terms of heat stress. In addition, the holdase chaperone function of IbOr protein, which regulates IbPsbP stability, enhances the stabilization of PSII, and thereby confers heat stress tolerance in sweetpotato. Our results suggest that *Or* represents a promising candidate gene for use in genetic engineering to achieve higher levels of nutritional carotenoids and environmental stress tolerance in plants.

## Materials and Methods

### Plant Materials and Growth Conditions

*Arabidopsis thaliana* ecotype Colombia-0 was used as the wild type (WT) for all experiments. *Arabidopsis* transgenic lines overexpressing empty vector (EV/At) or IbOr (*IbOr-OX*/At) were generated as described previously (Park et al., 2016), and grown in a growth chamber at 22 ± 1°C under a photoperiod of 16 h light/8 h dark.

Transgenic sweetpotato plants [*Ipomoea batatas* (L.) Lam. cv. Sinzami] overexpressing empty vector (EV/Ib) or IbOr (*IbOr-OX*/Ib) were generated as described previously (Park et al., 2015). Sweetpotato plants were propagated from stem cutting, and grown in a growth chamber at 25 ± 1°C under a photoperiod of 16 h light/8 h dark. Sweetpotato calli were subcultured on MS1D medium [0.43% basic salt Murashige & Skoog medium supplemented with 1 mg L^-1^ 2,4-dichlorophenoxyacetic acid (2,4-D), 3% sucrose, and 0.4% Gelrite] at intervals of 2 weeks, and samples were taken after two rounds of propagation.

### Stress Treatments

For two-dimensional electrophoresis (2-DE) analysis, transgenic *Arabidopsis* lines (EV/At, *IbOr-OX*/At) were cultured for 7 days on 1/2 MS medium (0.23% Murashige & Skoog medium including vitamins, 3% sucrose, and 0.6% phyto agar) in incubation chambers at 22 ± 1°C, followed by heat treatment at 42°C for 40 min. To investigate the expression of IbPsbP, 1-month-old sweetpotato plants with six fully expanded leaves were subjected to various stresses [38 and 47°C (heat), 4°C (cold), 100 mM methyl viologen (MV), 10 mM H_2_O_2_, and 200 mM NaCl] (Kim et al., 2014; Park et al., 2016), and then samples were taken from the fully expanded leaves third from the top of treated plants at the indicated time. Two-month-old sweetpotato plants were used for acquisition of samples of leaves from shoot apical meristem, according to position-dependent development stage (Ji et al., 2016). To test the abiotic stress tolerance of transgenic sweetpotato, 1-month-old *IbOr-OX*/Ib and EV/Ib plants with six fully expanded leaves were subjected to heat treatment at 47°C for 24 h, and then plants were recovered in a growth chamber at 25 ± 1°C. All samples were frozen in liquid nitrogen and stored at -80°C after processing. All experiments were repeated as three biological and three technical replicates.

### Protein Extraction, 2-DE, and Image Analysis

Total protein extractions were performed with trichloroacetic acid (TCA)/acetone, as previously described (Wang et al., 2008) with some modifications. *Arabidopsis* seedlings were ground in liquid nitrogen into a fine powder. Total protein was isolated from 200 mg of fine powder treated with 1.3 mL of cold TCA/acetone buffer (10% TCA and 0.07% 2-ME), mixed with SDS extraction buffer [30% sucrose, 2% SDS, and 0.1 M Tris-HCl (pH 8.8)] and saturated phenol, and finally precipitated by addition of 0.1 M ammonium acetate in methanol. The resultant protein was dissolved in lysis buffer [9 M urea, 4% CHAPS, 50 mM DTT, 1 mM PMSF, and 0.5% immobilized pH gradient (IPG) buffer (Amersham Biosciences, San Francisco, CA, United States)], vortexed for 1 min, and centrifuged for 1 h at 15°C, 40000 × *g*. The supernatant was collected and centrifuged again (15 min at 15°C, 40000 × *g*). Total protein was quantitated by the Bradford method (Kruger, 1994) using the Bio-Rad Quick Start^TM^ Bradford 1X Dye Reagent. Protein solutions were prepared and stored at -80°C. Aliquots (150 μg) of total protein were loaded on isoelectric focusing electrophoresis (IEF) gels. For first-dimension separation, IEF was carried out on a 17 cm long, pH 5–8, IPG strip on the Bio-Rad PROTEAN IEF system. Application settings were as follows: 250 V for 15 min, 10000 V for 3 h, and 80000 V for 8 h. Prior to sodium dodecyl sulfate–polyacrylamide gel electrophoresis (SDS-PAGE), the gel strips were reduced in equilibrating solution [30% glycerol, 50 mM Tris-HCl (pH 8.8), 6 M urea, 2% SDS] containing 1% DTT, and then alkylated with 2.5% iodoacetamide. Second-dimension SDS-PAGE separation was carried out on the Bio-Rad Protean II, with typical run conditions as follows: initial power, 2.5 W for 30 min, followed by 6 W/gel for 5–6 h in 13% resolving gel. After electrophoresis, proteins were visualized by silver staining. All experiments were repeated twice, and similar results were obtained.

Silver-stained gels were scanned on a GS-800 Imaging Densitometer (Bio-Rad, Hercules, CA, United States) at an optical resolution of 300 dpi, and the intensities of protein spots were calculated from scanned digitized images. 2-D gel electrophoresis data analysis software (Bio-Rad PDQuest 7.2.0; Bio-Rad, Hercules, CA, United States) was used for analysis of differentially expressed protein spots. After spots were detected and quantified, matching and editing were carried out. Silver-stained protein spots were de-stained, and in-gel trypsin digestion was carried out as described in Supplementary Material.

### RNA Preparation and Analysis of Gene Expression

Total RNA was extracted from the indicated plant tissues (*Arabidopsis* seedlings; sweetpotato callus, root, stem, and leaf) using the GeneAll Ribospin Plant^TM^ kit (GeneAll, Seoul, South Korea). For cDNA production, 2 μg of total RNA was reverse-transcribed to 20 μL of cDNA with 0.1 pmol of gene-specific primers using a RT-PCR kit (Enzynomics, Daejeon, South Korea). The reaction mixture was diluted 1:5 with sterilized water, and 2 μL of each reaction was subjected to real-time qRT-PCR. The gene-specific primers used in this study are listed in Supplementary Table S1. All quantitative RT-PCR (qRT-PCR) analyses were performed on a CFX real-time PCR system with the CFX system software (Bio-Rad) using Ever-Green 20 fluorescent dye (BioFACT, Daejeon, South Korea). The program used for PCR was as follows: initial denaturation for 15 min at 95°C, followed by 40 cycles of 95°C for 20 s, 60°C for 40 s, and 72°C for 30 s. Three biological repeats and three technical repeats were performed for each data point.

### Gene Cloning and Plasmid Construction

ORF sequences of IbPsbP and IbOr, containing the *attB* site and lacking a stop codon, were amplified with the indicated primers (Supplementary Table S1) to generate entry vectors based on pDONR207 using BP Clonase (Invitrogen, Carlsbad, CA, United States). For subcellular localization analysis, IbPsbP-PDONR207 was cloned into destination vector PGWB5 using LR Clonase (Invitrogen) to create the corresponding GFP fusion proteins. For bimolecular fluorescence complementation (BiFC), *IbPsbP* was transferred from the entry vectors to destination vector pDEST-VYNE (R) GW (containing the N-terminal fragment of Venus fluorescent protein), and IbOr was transferred from the entry vectors to destination vector pDEST-VYCE (R) GW (containing the C-terminal fragment of Venus fluorescent protein), using LR Clonase (Gehl et al., 2009). For protein expression, IbOr was transferred from the entry vectors to destination vector pDEST17 using LR Clonase to generate His-tag fusion proteins, and IbPsbP was transferred from the entry vectors to destination vector pDEST15 using LR Clonase to generate GST-tag fusion proteins.

### Phylogenetic Analysis

Sequences of PsbP family members were identified by BLAST searches in GenBank (UniProt and NCBI website). The published sequences were collated and converted to predicted amino acid sequences using BioEdit. Alignments were carried out using BioEdit and BoxShade server. Phylogenetic trees were constructed using Molecular Evolutionary Genetics Analysis Version 6. Neighbor-joining phylogenetic trees were created with 1,000 bootstrap replicates.

### Transient Expression in *N. benthamiana* and Confocal Microscopy

*Agrobacterium tumefaciens* GV3101 strains carrying the indicated constructs and P19-silencing suppressor gene cloned into pCAMBIA 1304 vector (Voinnet et al., 2003) were grown in YEP medium supplemented with the appropriate antibiotics overnight. Cultures were spun down and resuspended in infiltration solution (10 mM MES, 10 mM MgCl_2_, and 100 μM acetosyringone). The cultures were grown in infiltration solution to a final OD_600_ = 0.5. *Agrobacterium* carrying the indicated construct or combinations of constructs and P19 were co-infiltrated into 6-week-old *Nicotiana benthamiana* leaves for subcellular localization analysis. After 3 days of growth in a greenhouse at 25°C under LDs, the infiltrated parts of leaves were cut and subjected to fluorescence signal detection under a Leica TCs SP2 confocal microscope (Leica Microsystems, Heidelberg, Germany) with the appropriate filter sets, as described by Gehl et al. (2009).

### Protein Expression and Purification

For protein expression, *Escherichia coli* BL21 (DE3) cells were induced to express GST fusion proteins (GST-IbPsbP and GST-IbOr) or 6× His fusion proteins (His-IbPsbP and His-IbOr) by treatment with 0.5 mM isopropyl β-D-1-thiogalactopyranoside (IPTG) overnight at 15°C (Miroux and Walker, 1996; Park et al., 2016). The cells were disrupted by sonication and shaken rapidly on ice for 1 h after the addition of 1% Triton X-100. The samples were then centrifuged, and the supernatant was transferred to a new tube. The supernatants were incubated overnight with prewashed GST beads (GE Healthcare, Uppsala, Sweden) or Ni-NTA beads (Qiagen, Hilden, Germany) with gentle rotation at 4°C. The beads were extensively washed, and the fusion protein was eluted with 10 mM glutathione (Sigma–Aldrich, Ann Arbor, MI, United States) for GST-tag fusion proteins, or 250 mM imidazole (Fluka, Buchs, Switzerland) for His-tag fusion proteins (Ke et al., 2017). The concentration of each fusion protein was determined by the Bradford method, as described previously (Kruger, 1994).

### Bimolecular Fluorescence Complementation (BiFC) Assay

IbOr and IbPsbP were fused with Venus-C and Venus-N, respectively, using Gateway cloning technology. pDONR207-IbOr and pDONR207-IbPsbP were subjected to site-specific recombination into pVyCE and pVyNE, which contained the cauliflower mosaic virus 35S promoter and the C-terminal or N-terminal of Venus protein improved YFP derivative (Chen et al., 2008). Constructs were transformed into *A. tumefaciens* EHA105, and *Agrobacterium*-mediated transient expression was performed. Three days after infiltration, *N. benthamiana* leaves were cut off into small squares. The samples were examined for Venus fluorescence by confocal microscopy.

### Determination of Holdase Chaperone Activity

*In vitro* holdase chaperone activity was evaluated using GST:IbPsbP as substrates. The substrates were incubated in 50 mM HEPES-KOH (pH 8.0) buffer (Park et al., 2016) at 45 or 50°C with the indicated concentrations of His:IbOr. Substrate stability was determined by SDS-PAGE and western blotting using the appropriate antibodies.

*In vivo* holdase chaperone activity was evaluated using GFP:IbPsbP as substrates. Through Gateway cloning system, IbPsbP-pDONR207 and IbOr-pDONR207 were subjected to site-specific recombination into pGWB5 (35S promoter, C-sGFP) and pGWB11 (35S promoter, C-FLAG), respectively. IbPsbP and IbOr were fused with GFP and FLAG. GUS was amplified from the pENTE^TM^ GUS vector (Invitrogen, Carlsbad, CA, United States), after the PCR product was cloned into T-blunt and sequenced, clones were ligated into the pCAMBIA1300-multi vector by digestion with restrictive enzyme. All three plasmid constructs were transformed into *A. tumefaciens* strain GV3101 and transient expressed in *N. benthamiana* with or without FLAG:IbOr (as shown in **Figure 6B**). Infected part of tobacco leaves were used for total proteins isolation and western blotting with corresponding antibodies.

### Analysis of Photosynthetic Activity and Chlorophyll Contents

Photosynthetic activity in leaves was evaluated based on chlorophyll fluorescence determination of photochemical yield (Fv/Fm), which represents the maximal yield of the photochemical reaction in PSII (Butler and Kitajima, 1975), using a portable chlorophyll fluorescence meter (Handy PEA, Hansatech, England) after 30 min of dark adaptation. Chlorophyll contents were measured with a portable chlorophyll meter (SPAD-502, Konica Minolta, Japan). Total chlorophyll contents after stress treatment were compared with those under normal conditions. Both of these values were detected using the third through fifth intact, fully expanded leaves (counting from shoot apical meristem) of individual plants.

## Results

### Identification of Differentially Expressed Proteins

Our previous results suggested that IbOr plays important roles in protecting photosynthesis from abiotic stress via regulation of carotenoid biosynthesis and stabilization of PSII (Kim et al., 2013; Park et al., 2015, 2016). However, no photosynthetic proteins have been reported to interact with IbOr. To identify proteins that bind IbOr, we incubated 7-day-old transgenic *Arabidopsis* seedlings (EV/At and *IbOr-OX*/At) at 42°C for 40 min, and then extracted total proteins from the heat-stressed seedlings and subjected the samples to 2-DE analysis. We detected around 850 and 875 proteins in EV/At and *IbOr-OX*/At seedlings, respectively (Supplementary Figure S1).

**FIGURE 1:**
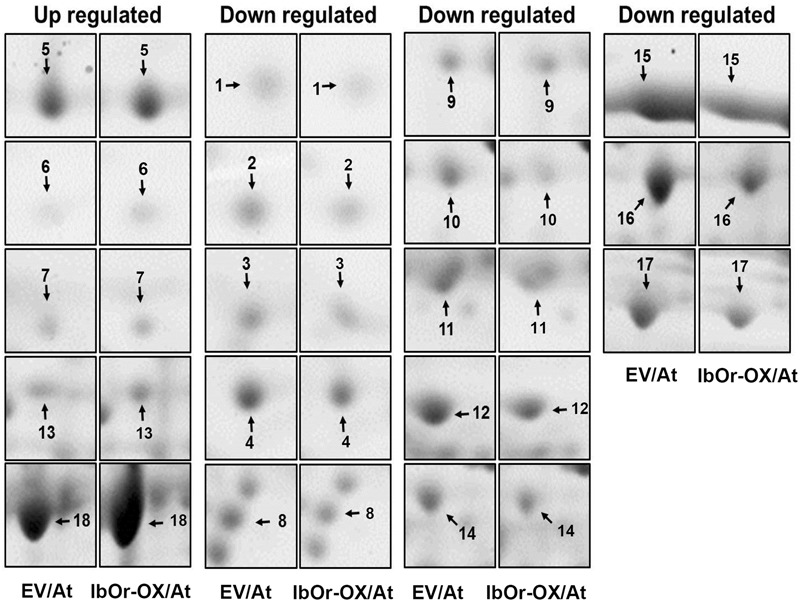
Enlarged views of 2-DE maps of up- or down-regulated proteins marked in Supplementary Figure S1. Spot numbers indicate proteins that were up-regulated or down-regulated in *IbOr-OX*/At vs. EV/At.

In total, we identified 18 protein spots that were reproducibly differentially expressed in *IbOr-OX/*At transgenic *Arabidopsis* seedlings (Supplementary Figure S1B) in comparison with EV (Supplementary Figure S1A). Among them, five proteins were up-regulated and 13 were down-regulated in *IbOr-OX/*At transgenic plants (**Figure 1**). All protein spots exhibiting statistically significant differences between EV and *IbOr-OX/*At transgenic plants were selected for further characterization. To investigate the functions of these proteins, we excised all 18 protein spots from silver-stained gels and digested them with trypsin, and subjected the digestion products to MALDI-TOF/TOF-MS analysis. The list of differentially expressed proteins is summarized in **Table 1**. SPX domain-containing protein 3 (Uni), oxygen-evolving enhancer protein 2-1 (PsbP), thioredoxin reductase 1, fructose-bisphosphate aldolase, and ribulose bisphosphate carboxylase large chain were up-regulated by 1.75-, 2.82-, 2.97-, 2.7-, and 2.3-fold, respectively. To investigate whether IbOr, which is involved in resistance to heat stress, can directly interact with these up-regulated proteins, we performed yeast two-hybrid assays. Among five up-regulated proteins, PsbP protein only interacted with IbOr (Supplementary Figure S2). Therefore, we focused on PsbP in our subsequent investigation of how IbOr regulates heat stress tolerance and photosynthesis. PsbP is an extrinsic subunit of PSII and participates in the normal function of photosynthetic water oxidation (Ifuku, 2014). However, its physiological roles have not been well-defined in higher plants.

**Table 1:** Identification of differentially expressed proteins in EV and *35S-IbOr Arabidopsis* transgenic seedlings treated with heat stress.

No.^a^	Description	ID (UniProt)	Mr/pI^b^ (Theoretical)	M.P.^c^	S.C. (%)^d^	Fold change^e^
1	17.4 kDa class III heat shock protein	Q9SYG1	17352/7.88	3	16	-3.00
2	Ribulose bisphosphate carboxylase large chain	K4ET10	20455/6.15	1	7	-2.11
3	Ribose 5-phosphate isomerase A	Q9S726	29401/5.72	6	32	-1.77
4	Glutathione *S*-transferase F2	P46422	24114/5.92	3	20	-1.80
5	SPX domain-containing protein 3	Q5PP62	28246/8.42	3	25	+1.75
6	Oxygen-evolving enhancer protein 2-1, chloroplastic	Q42029	28095/6.90	3	19	+2.82
7	Thioredoxin reductase 1	Q39243	35520/5.82	3	17	+2.97
8	Isoflavone reductase homolog P3	P52577	33773/5.66	4	16	-2.00
9	Thioredoxin reductase 2	Q39242	40895/6.26	8	26	-1.88
10	Ferredoxin-NADP reductase, leaf isozyme 2	Q8W493	41484/8.51	8	23	-3.76
11	Sedoheptulose-1,7-bisphosphatase	P46283	42787/6.17	10	28	-1.60
12	Photosystem II stability/assembly factor HCF136	O82660	44133/6.79	14	40	-2.08
13	Fructose-bisphosphate aldolase	Q9LF98	38858/6.05	10	39	+2.70
14	Probable cinnamyl alcohol dehydrogenase 9	P42734-2	33631/7.10	8	36	-2.03
15	Ribulose bisphosphate carboxylase/oxygenase activase	F4IVZ7	48754/7.55	8	29	-2.07
16	Cytosolic isocitrate dehydrogenase	Q9SRZ6	46059/6.13	13	35	-2.03
17	ATP synthase subunit alpha	P56757	55351/5.19	17	35	-2.06
18	Ribulose bisphosphate carboxylase large chain	O03042	53435/5.88	17	38	+2.30

*^a^Protein numbers (1–18) are consistent with those in **Figure 1** and Supplementary Figure S1. ^b^Theoretical MW (kDa) and pI values. ^c^Number of matched peptides. ^d^Percentage of sequence coverage. ^e^‘+’ up-regulation; ‘-’ down-regulation.*

### Isolation and Characterization of the Sweetpotato *PsbP* Gene (*IbPsbP*)

**FIGURE 2:**
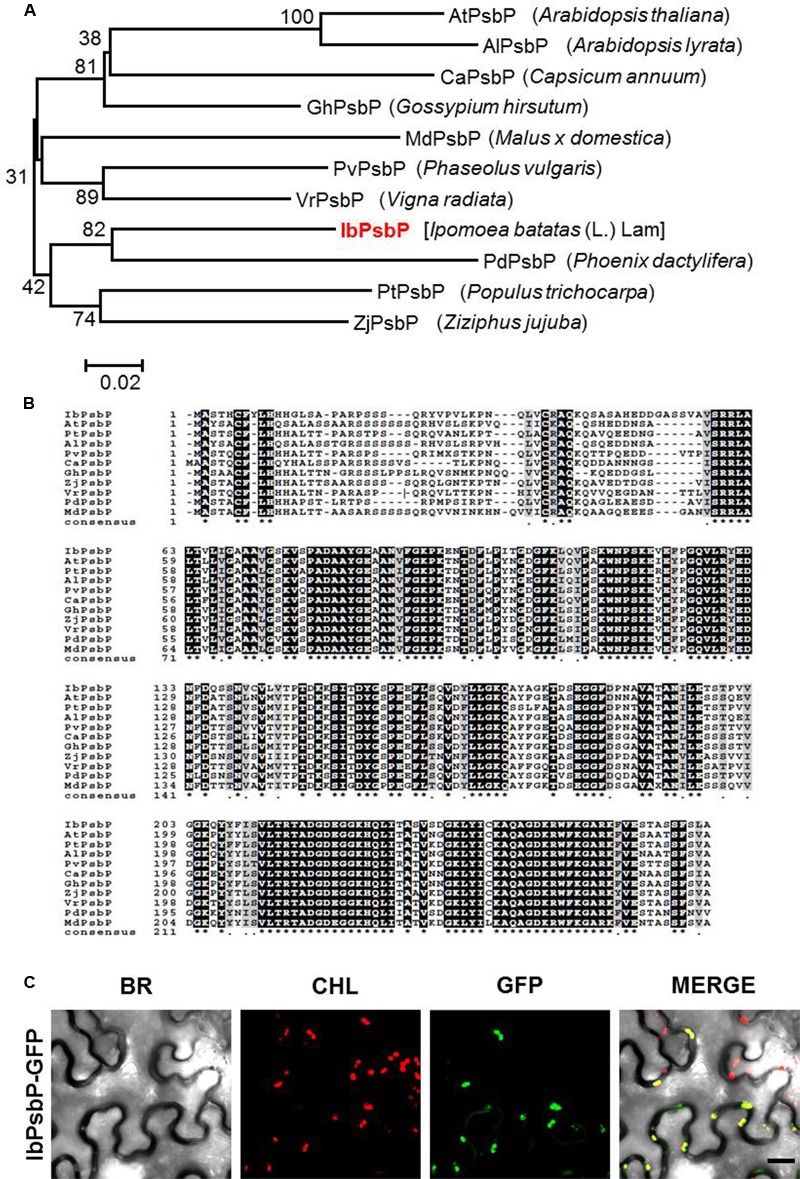
Amino acid sequence analysis of *IbPsbP*. **(A)** Phylogenetic analysis of *IbPsbP* in relation to other plant *PsbPs.* The phylogenetic tree was generated by the maximum-likelihood method using the MEGA software. **(B)** Alignment of the predicted amino acid sequence of *IbPsbP* with those of closely related *PsbPs*. Conserved amino acid residues are shaded in black. Alignment was performed using the CLUSTALW (1.82) multiple sequence alignment program (http://www.genome.jp/). The GenBank accession numbers for the PsbP subfamily are as follows: IbPsbP (KT873844), AtPsbP (Q42029), PtPsbP (A9PHM0), AlPsbP (D7LC89), PvPsbP (T2DP14), CaPsbP (A0A1U8FMI4), GhPsbP (A0A1U8PJK1), ZjPsbP (XP_015880618), VrPsbP (A0A1S3VW33), PdPsbP (XP_008809743), and MdPsbP (XP_008342330). **(C)** Subcellular localization of IbPsbP-GFP fusion protein. GFP-fused IbPsbP was transiently expressed in *Nicotiana benthamiana* leaves by agroinfiltration and observed by laser scanning confocal microscopy. Green fluorescence of IbPsbP-GFP was detected in chloroplasts. BR, bright field microscopy images; CHL, chloroplast autofluorescence images; GFP, GFP fluorescence images; MERGE, overlay images of bright field, chloroplast, and GFP fluorescence images. Scale bar = 20 μm.

We isolated the cDNA fragment of *IbPsbP* from orange-fleshed sweetpotato (cv. Sinhwangmi) by homology-based BLAST searches against the sweetpotato transcriptome database (unpublished data). Phylogenetic analysis of the deduced plant PsbP amino acid sequences from 11 species indicated that IbPsbP clusters closely with other PsbP homologs (**Figure 2A**). The predicted protein IbPsbP contains 268 amino acids, and comparison of the deduced amino acid sequence revealed that IbPsbP shares 73–80% amino acid identity with other PsbP homologs (**Figure 2B**). These results suggest that IbPsbP shares a conserved role with other PsbP homologs.

To investigate the subcellular localization of IbPsbP, we transiently expressed GFP-fused IbPsbP in *N. benthamiana* leaves by Agro-infiltration. Three days after infiltration, the epidermal cells of infiltrated leaves were observed by confocal laser scanning microscopy. As shown in **Figure 2C**, GFP fluorescence produced by IbPsbP-GFP fusion proteins overlapped with chlorophyll fluorescence, indicating that IbPsbP is a chloroplast protein.

### Expression Profile of IbPsbP

**FIGURE 3:**
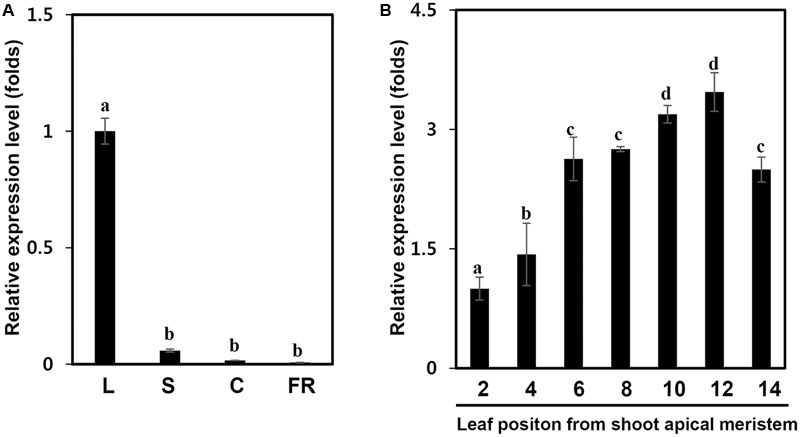
*IbPsbP* expression profiles in different sweetpotato tissues and leaf development stage. **(A)** *IbPsbP* expression profiles in different sweetpotato tissues. The leaf, stem, and fibrous root of 1-month-old sweetpotato plants and calli subcultured at 2 week intervals were used to analyze *IbPsbP* expression. L, the third expanded leaf from the top of the sweetpotato plant; S, stem; C, calli; F, fibrous root. **(B)** *IbPsbP* gene expression during position-dependent leaf development in sweetpotato. Leaves from shoot apical meristem of 2-month-old sweetpotato plants were used to analyze *IbPsbP* expression. Data represent the means ± SD from five independent plants. Within columns, letters followed by the same letter indicate no significant difference (Duncan’s multiple range test, *P* < 0.05).

We next examined the expression of the *IbPsbP* gene in various tissues of sweetpotato. *IbPsbP* transcripts were most abundant in leaves among the tissues examined (leaf, stem, and fibrous root) of 1-month-old sweetpotato plants and in calli (**Figure 3A**). We then investigated *IbPsbP* gene expression at every position along the leaf (from shoot apical meristem) of 2-month-old sweetpotato plants. As shown in **Figure 3B**, *IbPsbP* was expressed preferentially in older leaves (most abundant in leaf position 12) in comparison with tender leaves.

**FIGURE 4:**
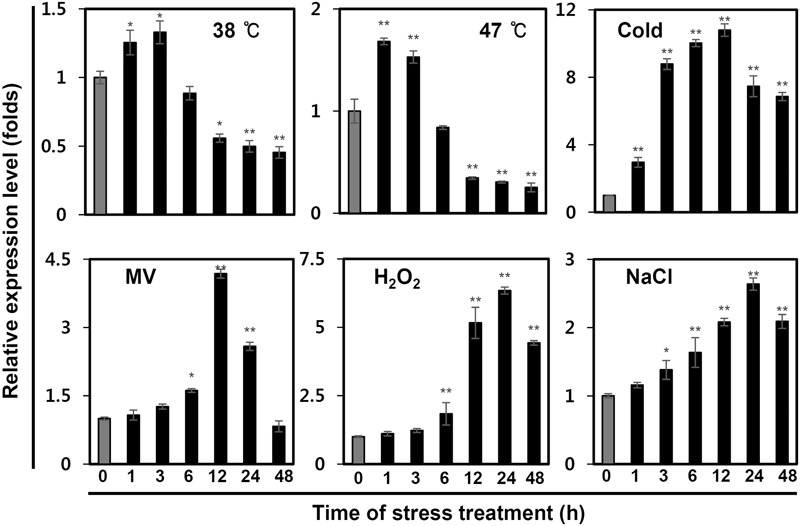
*IbPsbP* expression profiles in sweetpotato leaves under various stresses. One-month-old sweetpotato plants were treated with heat, cold, MV, H_2_O_2_, or NaCl, and then the third expanded leaves from the tops of treated plants were used to analyze *IbPsbP* expression. Cold stress treatment was carried out at 4°C; 100 mM MV, 10 mM H_2_O_2_, and 200 mM NaCl were used for other stress treatments. Data represent three independent experiments. Asterisks indicate significant differences: ^∗^*P* < 0.05 or ^∗∗^*P* < 0.01 (*t*-test).

In addition, we investigated whether *IbPsbP* transcripts levels were affected by multiple abiotic stresses, including heat (38 and 47°C), cold (4°C), MV (100 μM), H_2_O_2_ (10 mM), and salt (200 mM NaCl). For this purpose, we continually monitored *IbPsbP* expression in the third expanded leaves from treated plants over 48 h. As shown in **Figure 4**, *IbPsbP* was induced by multiple abiotic stresses, but the expression pattern differed depending on the specific nature of the stress. *IbPsbP* transcript levels were elevated 1.4- and 1.7-fold in the early phase of heat stress. *IbPsbP* was also up-regulated fourfold at 12 h by MV treatment and reached maximum expression (2.6-fold) at 24 h by NaCl treatment. Interestingly, *IbPsbP* was dramatically induced after 3 h of cold treatment and reached the highest expression level (10.7-fold) at 12 h, followed by a decrease. In addition, *IbPsbP* was up-regulated sixfold by H_2_O_2_ treatment (**Figure 4**). These results suggest that *IbPsbP* transcripts are tissue-specifically expressed, and may play an important role in response to abiotic stress in sweetpotato plants.

### IbOr Holdase Chaperone Activity Stabilizes IbPsbP

**FIGURE 5:**
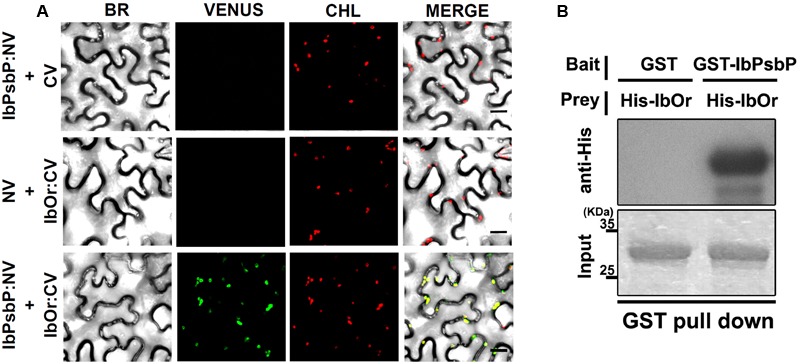
IbPsbP interacts with IbOr. **(A)** Bimolecular fluorescence complementation (BiFC) assays for *in planta* interaction of IbPsbP with IbOr in chloroplasts. *N. benthamiana* leaves were transformed by *Agrobacterium* harboring N-terminal region of Venus (NV) and C-terminal region of Venus (CV) construct pairs and observed by confocal laser scanning microscopy. BR, bright field microscopy images; CHL, chlorophyll autofluorescence; VENUS, Venus fluorescence images; MERGE, overlay of bright field, chlorophyll, and Venus images. Scale bar = 20 μm. **(B)** Pull-down assay for *in vitro* interaction of IbOr with IbPsbP. Gels containing pull-down assay products were immunoblotted with anti-His. His-IbOr, GST (negative control), and GST-IbPsbP proteins are shown in the indicated combinations.

Our previous results indicated that IbOr functions as a holdase chaperone to stabilize phytoene synthase (IbPSY) in sweetpotato (Park et al., 2016). To determine whether IbOr plays a similar role in protecting IbPsbP from heat stress induced denaturation, we first examined the interaction between IbOr and IbPsbP in plant cells using BiFC assays. Cell suspensions of *A. tumefaciens* carrying constructs encoding N-terminal Venus fluorescent protein NV-IbPsbP and C-terminal Venus fluorescent protein CV-IbOr were infiltrated into *N. benthamiana* leaves. We detected strong Venus fluorescence in chloroplasts when NV-IbPsbP and CV-IbOr were used in combination indicating that IbOr interacts with IbPsbP, predominantly in chloroplasts (**Figure 5A**). This interaction was confirmed by *in vitro* pull-down assays (**Figure 5B**).

**FIGURE 6:**
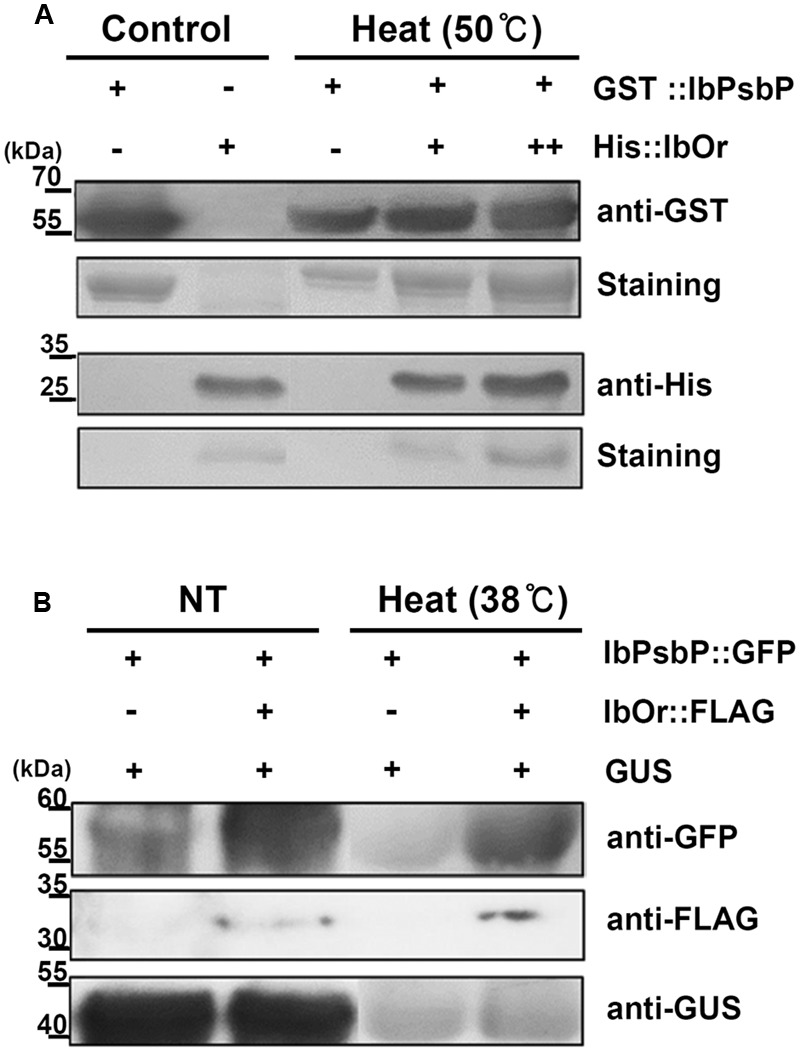
IbOr holdase chaperone activity for IbPsbP. **(A)** IbOr regulates IbPsbP stability under heat stress *in vitro*. Equal amounts of purified IbPsbP were incubated at 50°C for 30 min, with or without IbOr. GST-IbPsbP fusion protein and His-IbOr fusion protein levels were detected by Coomassie brilliant blue staining of 12% SDS-PAGE gels (GST-IbPsbP, the second row; His-IbOr, the fourth row), followed by western blotting with the indicated antibodies (anti-GST, the first row; anti-His, the third row). Purified IbPsbP and IbOr proteins were loaded as controls. **(B)** IbOr regulates IbPsbP stability under heat stress *in planta*. Constructs containing indicated combinations were transiently overexpressed in 3-week-old *N. benthamiana* leaves by *Agrobacterium* infiltration. Heat stress (in 38°C for 1 h) were treated at 3 days after infiltration. Total proteins were extracted from infected leaves and followed by western blotting with the indicated antibodies (anti-GFP, the first row; anti-FLAG, the second row; anti-GUS, the third row). GUS protein were used as expression control.

We next tested whether the IbOr–IbPsbP complex affects the stability of IbPsbP upon heat stress. To this end, we purified bacterially expressed recombinant GST:IbPsbP protein and evaluated its stability under heat stress. Specifically, GST:IbPsbP was incubated in the presence or absence of purified recombinant His:IbOr protein at the indicated temperatures, and then IbPsbP levels were analyzed on SDS-PAGE by immunoblotting. Previous work showed that IbOr is stable even at 70°C (Park et al., 2016), whereas GST:IbPsbP protein aggregated at 50°C after 30 min. However, when purified recombinant His:IbOr protein was included in Tris-HCl (pH 8.0) buffer, IbPsbP was protected from aggregation (**Figure 6A**). These results indicated that IbOr holdase chaperone activity can stabilize IbPsbP during heat stress *in vitro*. To confirm IbOr holdase chaperone activity for IbPsbP *in planta*, further investigation was carried out. *A. tumefaciens* carrying GFP:IbPsbP fusion protein and the expression control GUS protein with or without FLAG:IbOr fusion protein were transiently co-expressed in well-conditioned *N. benthamiana* in a consistent state. Three days after infiltration, infiltrated plants were subjected to heat stress at 38°C for 1 h. Total proteins were isolated from infected leaves before and after heat treatment, and were analyzed by western blotting with anti-GFP, anti-FLAG, anti-GUS. GFP:IbPsbP protein shows parallel stability in the presence or absence of FLAG:IbOr fusion protein co-expression under normal condition (**Figure 6B**). However, after heat treatment (38°C, 1 h), GFP:IbPsbP protein was severely degraded in infiltrated plants without FLAG:IbOr protein, but was fairly consistent with FLAG:IbOr protein co-expression (**Figure 6B**). Taken together, these results indicated that IbOr holdase chaperone activity can stabilize IbPsbP under heat stress condition *in planta*.

### Overexpression of IbOr Confers Tolerance of Heat Stress in Transgenic Sweetpotato Plants

**FIGURE 7:**
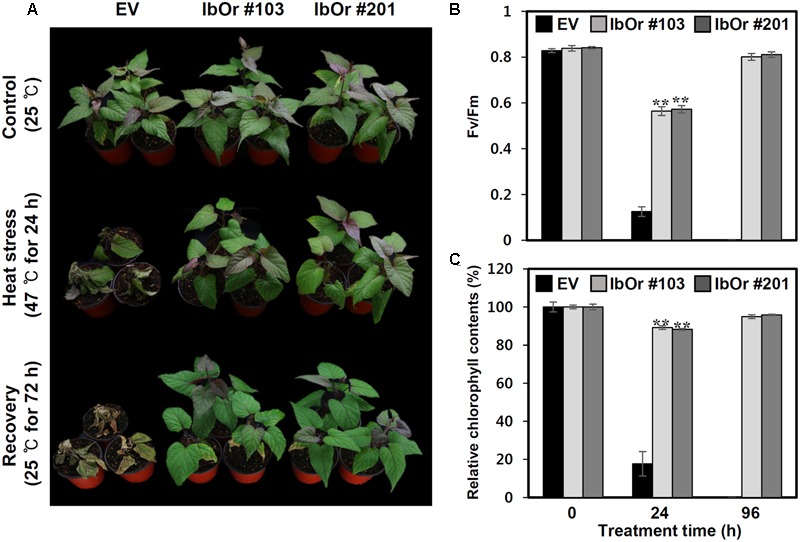
Heat stress analysis of IbOr overexpressing transgenic sweetpotato. 1-month-old sweetpotato plants were subjected to heat stress (47°C) for 24 h, followed by 72 h recovery. Three biological and three technical replicates were performed for each data point. **(A)** Phenotypes of 1-month-old EV and IbOr plants before and after heat treatment. **(B)** Fluorescence-based maximum quantum yield of PSII (Fv/Fm) and **(C)** relative chlorophyll contents (Chl) in the third leaves of EV and IbOr plants after 24 h heat stress and 72 h recovery. Data represent three independent experiments. Asterisks indicate significant differences: ^∗∗^*P* < 0.01 (*t*-test).

In higher plants, PsbP protein is essential for the regulation and stabilization of PSII, and is required for normal thylakoid architecture (Yi et al., 2007, 2009). Heat or oxidative stress induced damage frequently occurs in the OEC of PSII (Allakhverdiev et al., 2008). To determine whether IbOr overexpression confers increased PSII efficiency upon exposure to heat stress, we developed transgenic sweetpotato plants harboring empty vector (EV/Ib) or IbOr overexpression construct (*IbOr-OX*/Ib), and then subjected 1-month-old pot-cultured EV/Ib and *IbOr-OX*/Ib plants of the same health status to high-temperature (47°C) treatment. Because oxygen-evolving activity of PSII started to decline at 40°C with half inactivation at 46–47°C (Allakhverdiev et al., 2007), we treated the EV/Ib and *IbOr-OX*/Ib plants with heat stress at 47°C. After 24 h of heat stress, EV/Ib plants exhibited wilting and chlorosis, whereas *IbOr-OX*/Ib plants grew well and exhibited only slight yellowish leaf coloration (**Figure 7A**). *IbOr-OX*/Ib plants maintained approximately 4.6- and 5.1-fold higher PSII efficiency and chlorophyll content, respectively, than EV/Ib plants (**Figures 7B,C**), consistent with their high temperature resistant phenotype. These results indicate that the holdase chaperone function of IbOr, which regulates IbPsbP stability, stabilizes PSII, thereby conferring heat stress tolerance on sweetpotato plants.

## Discussion

Or, a multi-functional protein, plays important roles in the regulation of carotenoid biosynthesis (Li et al., 2001; Lu et al., 2006; Lopez et al., 2008) and the environmental stress response in plants. Although its roles in carotenoid accumulation have been extensively studied in plants, elucidation of the mechanisms underlying the Or-mediated environmental stress response would greatly accelerate current plant-breeding programs.

2-DE combined with mass spectrometry is the most widely used approach for comparing plant proteomes with the goal of identifying differentially expressed proteins. In particular, this strategy has been employed to analyze changes in protein expression in response to environmental changes. Previously, we identified and characterized the gene encoding the Or ortholog (*IbOr*) from sweetpotato (Kim et al., 2013). Overexpression of *IbOr* results in accumulation of carotenoids and maintenance of higher PSII efficiency upon exposure to abiotic stress (particularly heat stress) in sweetpotato calli, *Arabidopsis*, alfalfa, and potato (Kim et al., 2013; Goo et al., 2015; Wang et al., 2015; Park et al., 2016). These results suggest that IbOr might play important roles in protecting photosynthesis against abiotic stress via regulation of carotenoid biosynthesis and stabilization of PSII.

In this study, we performed a comparative proteomic study of heat-treated transgenic *Arabidopsis* seedlings (EV/At and *IbOr-OX*/At) to identify proteins differentially expressed due to IbOr overproduction. Among 18 differentially expressed proteins, two (#1 and #13) participated in light-dependent reactions and the Calvin cycle, respectively (**Table 1**) (Flechner et al., 1999; Nishizawa et al., 2006). These results indicated that IbOr might be directly involved in the regulation of photosynthesis. Oxygen-evolving enhancer protein 2-1 (PsbP), an extrinsic protein of the OEC of PSII (Roose et al., 2007), was up-regulated in heat-treated *IbOr-OX*/At seedlings. Previous studies revealed that PsbP is essential for the regulation and stabilization of PSII, and is also required for normal thylakoid architecture in higher plants. In transgenic *Arabidopsis* and tobacco plants, down-regulation of wild PsbP by RNA interference led to retarded growth, pale green leaves, reduced PSII activity, and severe alterations in the PSII core protein complement. It remains unknown whether IbOr participates in photosynthesis through stabilization of PsbP.

In this study, we identified the PsbP homolog (*IbPsbP*) in sweetpotato plants. At the amino acid sequence level, *IbPsbP* clusters closely with other PsbP homologs, and its transcript is expressed specifically in young leaves. PSII is one of the predominant macromolecular assemblies of the photosynthetic apparatus in both cyanobacteria and higher plants. Biogenesis of PSII requires the coordinated assembly of nuclear- and chloroplast-encoded protein subunits. We found that IbPsbP predominantly localized to the chloroplast, consistent with the notion that PsbP is a nuclear-encoded thylakoid luminal-localized protein in higher plants and green algae (De Las Rivas et al., 2007; Ishihara et al., 2007; Liu et al., 2012). Furthermore, OEC proteins are membrane-extrinsic luminal subunits of PSII, and sensitive to abiotic stress (especially light stress) (Pérez-Bueno et al., 2011). The extrinsic protein PsbP is a subunit of the OEC, and its transcripts and protein levels are altered under abiotic stress in various plant species (Pérez-Bueno et al., 2004; Ifuku et al., 2008). In this study, we observed that IbPsbP transcript levels were elevated to varying degrees upon exposure to various stresses, including heat, cold, salt, and oxidative stress (**Figure 4**), consistent with previous studies. These results provide evidence that IbPsbP is functionally conserved among plant species. Interestingly, *IbPsbP* is dramatically induced by cold and oxidative stresses. Previous studies demonstrated that *PsbP* gene was induced by chilling stress in sugarcane (Huang et al., 2012), whereas *PsbP* were significantly down-regulated in rice and poplar plants subjected to cold treatment (Song et al., 2013; Maruyama et al., 2014). These results indicated that PsbP has variable functions in response to cold stress between different plant species. In addition, thermal stress induced the production of H_2_O_2_, and thereby caused oxidative damage in photosynthetic machinery (Allakhverdiev et al., 2008). PsbU, instead of PsbP, have enhanced mechanisms to detoxify exogenously applied H_2_O_2_ in cyanobacteria (Balint et al., 2006). Further physiological experiments are necessary to clarify whether the PsbP protein plays additional roles in response to cold and oxidative stresses. Although several studies regarding the protecting function of PsbP on PSII under unsuitable environmental conditions have been published, the biochemical features of IbPsbP remain to be elucidated (Pérez-Bueno et al., 2004; Ifuku et al., 2008).

In *Arabidopsis*, Or family proteins interact directly with PSY and function as the major regulators of active PSY protein abundance in carotenoid biosynthesis (Zhou et al., 2015). Our previous studies revealed that IbOr functions as a molecular chaperone to stabilize IbPSY, leading to carotenoid accumulation (Park et al., 2016). In addition, IbOr protein normally localizes in nucleus and plastids, but it translocates from nucleus to chloroplast upon exposure to heat stress (Park et al., 2016). At present, however, little is known about the translocation mechanism of IbOr. In this study, our BiFC experiments revealed that IbOr interacts with IbPsbP in chloroplasts (**Figure 5A**), and this IbOr–IbPsbP complex protects IbPsbP from aggregation under heat stress (**Figure 6**). These results suggest that IbOr might also function as a holdase chaperone to stabilize IbPsbP upon exposure to abiotic stress. Because photosynthesis is sensitive to heat stress, in order to examine photosynthetic efficiency, we measured the fluorescence-based maximum quantum yield for PSII (Fv/Fm) and relative chlorophyll contents of *IbOr* transgenic sweetpotato plants under heat stress. *IbOr* transgenic sweetpotato plants had much higher Fv/Fm and more stable relative chlorophyll content than plants harboring empty vector, which indicated that heat tolerance was enhanced in *IbOr* transgenic sweetpotato plants (**Figure 7**). In addition, the elevated heat stress tolerance in *IbOr* transgenic sweetpotato plants may be related to stabilization of IbPsbP, which contributes to homeostasis of the photosystem and further improves stress tolerance. These results are consistent with observations that overexpression of IbOr in *Arabidopsis*, alfalfa, and potato results in maintenance of higher PSII efficiency in response to abiotic stress. To further investigate the function of IbPsbP in PSII of sweetpotato, we developed transgenic sweetpotato plants in which IbPsbP was either overexpressed or down-regulated. In the future, the performance of these transgenic sweetpotato plants should be characterized to determine the molecular mechanisms of IbPsbP.

## Conclusion

**FIGURE 8:**
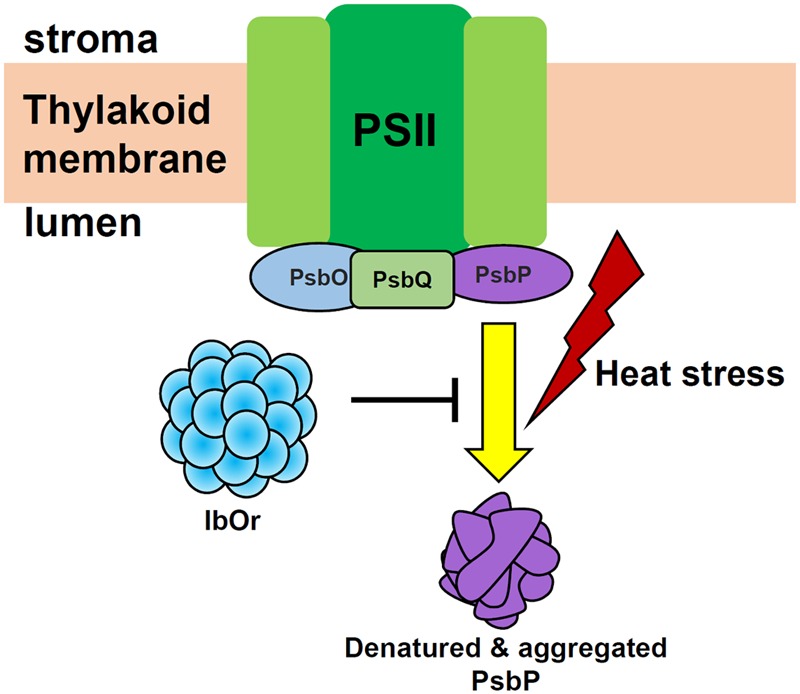
Proposed model of the function of IbOr in photosynthesis. When plants are exposed to heat stress, IbOr holdase chaperone activity is required to prevent IbPsbP aggregation. IbOr-mediated protection of IbPsbP increases tolerance of heat stress.

As shown by the model in **Figure 8**, this study was the first examination of the interaction between IbOr (a chaperone protein) and IbPsbP (an extrinsic protein of OEC), and the results reveal that IbOr might participate in photosynthesis in sweetpotato by stabilizing IbPsbP stability. Together with post-transcriptional regulation of IbPSY (the rate-limiting enzyme in the carotenoid biosynthetic pathway), IbOr as multi-functional protein, has tremendous potential for carotenoid accumulation and environmental stress tolerance in sweetpotato plant. Thus, *IbOr* represents a promising candidate gene for use in genetic engineering aimed at increasing nutritional value and environmental tolerance in plants, and the resulting transgenic crops could then be grown on marginal lands around the world to aid in sustainable development.

## Author Contributions

LK, HK, and S-SK conceived and designed the experiments. LK, HK, YK, QK, CJ, and S-CP performed the experiments. LK, HK, and QK analyzed the data. H-SL and S-SK contributed reagents/materials/analysis tools. LK, HK, and S-SK wrote the paper.

## Conflict of Interest Statement

The authors declare that the research was conducted in the absence of any commercial or financial relationships that could be construed as a potential conflict of interest.
