# Supplementary material for: IbOr Regulates Photosynthesis under Heat Stress by Stabilizing IbPsbP in Sweetpotato
Source: Front Plant Sci. 2017 Jun 8;8:989. doi: 10.3389/fpls.2017.00989 (PMC5462972; doi:10.3389/fpls.2017.00989)
Supplement: Supplementary file 1 [file Data_Sheet_1.docx]

***Supplementary Material***

**IbOr Regulates Photosynthesis under Heat Stress by Stabilizing IbPsbP in Sweetpotato**

*Le Kang^1, 2, †^, Ho Soo Kim^1, †^, Young Sang Kwon^3^, Qingbo Ke^1^, Chang Yoon Ji^1, 2^, Seyeon Park^1, 2^, Haeng-Soon Lee^1, 2^, Xiping Deng^4^, Sang-Soo Kwak^1, 2*^*

*^1^Plant Systems Engineering Research Center, Korea Research Institute of Bioscience and Biotechnology (KRIBB), Daejeon, Korea*

*^2^Department of Green Chemistry and Environmental Biotechnology, Korea University of Science and Technology (UST), Daejeon, Korea*

*^3^Environmental Biology and Chemistry Center, Korea Institute of Toxicology, Jinju, Korea*

*^4^State Key Laboratory of Soil Erosion and Dryland Farming on the Loess Plateau, Institute of Soil and Water Conservation, Northwest A&F University, Shaanxi, China*

*Running title: IbOr regulates IbPsbP stability*

*^†^ These authors contributed equally to this work.*

****Correspondence:***

*Dr. Sang-Soo Kwak*

*E-mail: sskwak@kribb.re.kr*

*Plant Systems Engineering Research Center, Korea Research Institute of Bioscience and Biotechnology (KRIBB), Daejeon, Korea*

**1. Supplementary Materials and Methods**

**Trypsin digestion and MALDI-TOF/TOF-MS**

Silver-stained protein spots were de-stained, and in-gel trypsin digestion was carried out as previously described (Katayama et al., 2001; Lee et al., 2004). Briefly, the gel pieces were de-stained [with 15 mM Fe(CN)_6_ and 50 mM Na_2_S_2_O_3_ for a few minutes], and then washed five times with 500 μL of MWA solution (50% methanol : 40% water : 10% acetic acid) for 30 min. The gels were then mixed with 500 μL of 50 mM NH_4_HCO_3_ and 500 μL of ACN (5 min), and vacuum-dried. Reduction of the de-stained gel pieces was performed with a solution of 10 mM DTT/0.1 M NH_4_HCO_3_ for 45 min at 56 °C, and they were alkylated in 55 mM C_2_H_4_INO/0.1 M NH_4_HCO_3_ for 30 min at room temperature in the dark and completely vacuum-dried. Next, the dried gel pieces were re-hydrated in 3 μL of a digestion buffer (25 mM NH_4_HCO_3_, 0.1% n-octyl glucoside) containing 50 ng mL^-1^ trypsin. Post-rehydration, 5–15 μL of digestion buffer (minus the trypsin) was added to the gel pieces. Peptide extraction was performed twice with one volume of ACN/H_2_O/CF_3_COOH (66:33:0.1, v/v/v) solution, after which the sample was sonicated, centrifuged, and dried in a SpeedVac. The protein sample was dissolved in 50% ACN/0.1% TFA, and stored at -20°C until further use.

**Protein identification**

The digested peptide solution (above) was carefully spotted onto the MALDI-TOF/TOF target plate using a micro-pipette. Analysis was carried out on an ABI 4800 Plus TOF-TOF Mass Spectrometer (Applied Biosystems, Framingham, MA, USA). The running conditions were as follows: 200 Hz ND: 355 nm YAG laser operations; signal/noise ratio >25; 10 higher intense ions were used for MS/MS analysis in 1 kV mode, 1000–1250 consecutive laser exposure. Spectral data (MS and MS/MS) were unpacked using the NCBI GreenPlants database, the UniProt database, and the Protein Pilot V.3.0 database at a mass tolerance of 100 ppm. MS/MS spectra search criteria in the databases were as follows: single missing pick, oxidation of methionines, and carbamidomethylation of cysteines. A statistically significant threshold value of *p* < 0.05 was used for searching individual peptide ion scores.

**Yeast two-hybrid assay**

For yeast two-hybrid assays, all genes tested were cloned into two vectors (pGAD424 and pAS2-1) and co-transformed into yeast strain pJ69-4A. Transformation, yeast growth, and quantitative β-galactosidase assays were performed as described in CLONTECH Yeast Protocols. Transformed yeasts were grown on SD medium lacking Trp, Leu, and His and supplemented with 5 mM 3-aminotriazole or on medium lacking Trp and Leu.

**References**

Katayama, H., Nagasu, T., and Oda, Y. (2001). Improvement of ingel digestion protocol for peptide mass fingerprinting by matrix-assisted laser desorption/ionization time-of-flight mass spectrometry. *Rapid Commun. Mass Spectrom.* 15, 1416-1421. doi: 10.1002/rcm.379

Lee, K., Kye, M., Jang, J. S., Lee, O. J., Kim, T., and Lim D. (2004). Proteomic analysis revealed a strong association of a high level of α1-antitrypsin in gastric juice with gastric cancer. *Proteomics* 4, 3343-3352. doi: 10.1002/pmic.200400960

**2. Supplementary Figures and Tables**

**2.1 Supplementary Figures**


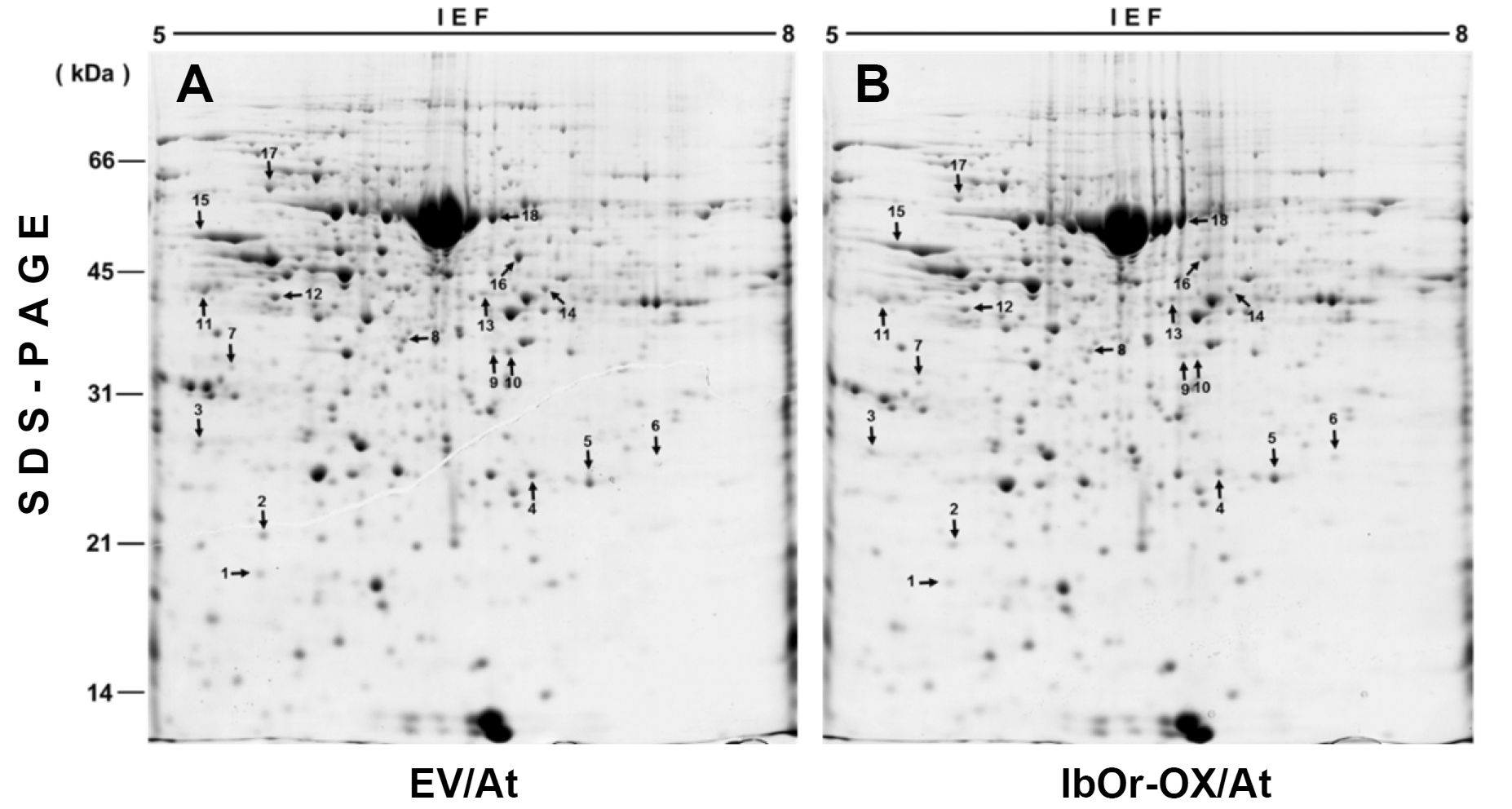


**Supplementary FIGURE S1 | High-resolution 2-DE image of total proteins extracted from heat stress–treated transgenic *Arabidopsis* seedlings harboring empty vector (A,** **EV/At) or overexpressing IbOr (B, *IbOr-OX*/At). (A)** Empty vector (EV/At). **(B)** IbOr-overexpressing transgenic *Arabidopsis* seedling (*IbOr-OX*/At).

**
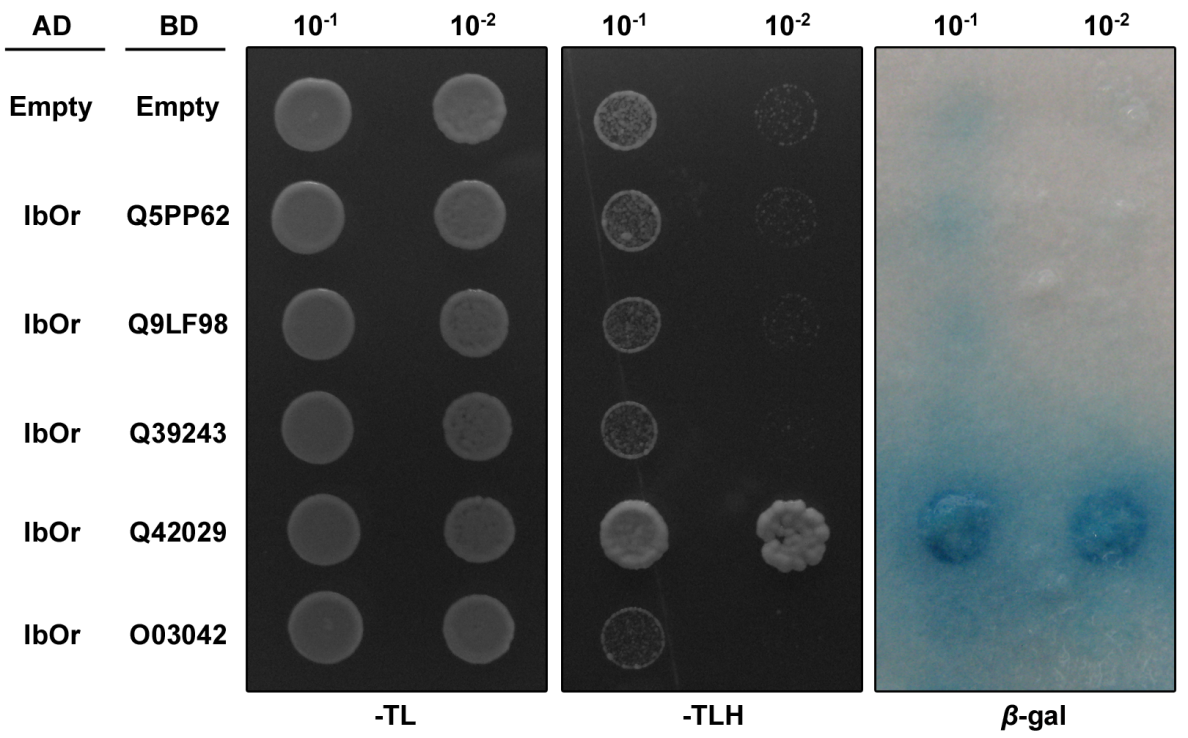
**

**Supplementary FIGURE S2 | Yeast two-hybrid assays of IbOr with up-regulated genes.** IbOr was fused to the activation domain (AD, pGAD424 vector), and up-regulated genes were fused to the binding domain (BD, pAS2-1 vector). Yeast cells transformed with combinations of constructs were spotted on minimal medium without Trp and Leu (-TL), as well as selective medium without Trp, Leu, and His (-TLH). *β*-galactosidase activity (*β*-gal) was detected by filter-lift assay. The GenBank accession numbers for the up-regulated genes are as follows: Q5PP62 (SPX domain-containing protein 3), Q9LF98 (fructose-bisphosphate aldolase), Q39243 (thioredoxin reductase 1), Q42029 (oxygen-evolving enhancer protein 2-1, chloroplastic), and O03042 (ribulose bisphosphate carboxylase large chain).

**2.2 Supplementary Table**

**Table S1. Gene-specific primer sequences used in this study**

| Gene | Sequence (5'-...-3') | | Application |
| --- | --- | --- | --- |
| *AttB1-IbPsbP-*F  *AttB2-IbPsbP-*R | | AAAAAGCAGGCTCAATGGCTTCCACACACTGC  AGAAAGCTGGGTTAGCAAGGCTGAAAGAACT | ORF cloning  ORF cloning |
| *AttB1-IbOr-*F  *AttB2-IbOr-*R | | AAAAAGCAGGCTCAATGGTATATTCAGGTAGAATCTTG  AGAAAGCTGGGTTATCAAATGGGTCAATTCGTGG | ORF cloning  ORF cloning |
| *pDONR207-*F  *pDONR207-*R | | TCGCGTTAACGCTAGCATGGATCTC  GTGTCTCAAAATCTCTGATGTTAC | RT-PCR  RT-PCR |
| *GFP-*F  *GFP-*R | | ATGAGTAAAGGAGAAGAACTT  GGCGCGCCTTTGTATAGT | RT-PCR  RT-PCR |
| *IbPsbP-*F  *IbPsbP-*R | | CCAGTGGTCGGAGGAAAGCA  CTGCCTGTGCCTTGCAGATG | qRT-PCR  qRT-PCR |
| *α-Tubulin-*F  *α-Tubulin-*R  *GUS-*F  *GUS-*R | | CAACTACCAGCCACCAACTGT  CAAGATCCTCACGAGCTTCAC  GGATCCATGTTACGTCCTGTAG  GTCGACTTATTGTTTGCCTCCCT | qRT-PCR  qRT-PCR  RT-PCR  RT-PCR |
|  | |  |  |
